# A rare case of large renal leiomyoma diagnosed histopathologically after surgical management: A case report

**DOI:** 10.1016/j.ijscr.2024.110429

**Published:** 2024-10-10

**Authors:** Anak Agung Ngurah Krisnanta Adnyana, Novitasari, I Wayan Suarsana, Ida Bagus Oka Widya Putra

**Affiliations:** a Wangaya General Hospital, Denpasar, Bali, Indonesia; b Faculty of Medicine, Mahasaraswati Denpasar University, Wangaya General Hospital, Denpasar, Bali, Indonesia; c Departement of Urology, Wangaya General Hospital, Denpasar, Bali, Indonesia

**Keywords:** Benign renal tumor, Case report, Nephrectomy, Radical nephrectomy, Renal leiomyoma

## Abstract

**Introduction:**

Renal leiomyoma is a rare renal tumor that originates from smooth muscle. Among all existing benign renal tumors, leiomyoma is one of the least common benign renal tumors.

**Case presentation:**

We report of a case report of a 43-year-old male complaints of palpable mass on the upper left abdomen, abdominal discomfort and hematuria. Contrast-enhanced CT scan revealed a solid heterogenous mass on the left kidney, adherent to the left abdominal wall and pushed the spleen cranially. Patient underwent radical nephrectomy and histopathology results revealed leiomyoma. Two weeks after surgery, the patient was asymptomatic.

**Discussion:**

Imaging of renal leiomyoma may provide a clue with a general finding of a well-defined tumor margin and no local invasion. In our case preoperative CT findings made the initial diagnosis inconclusive since it showed the renal mass was adherent to the abdominal wall. Definitive diagnosis was only possible through histopathologic examination.

**Conclusion:**

Radical nephrectomy remains as the mainstay of treatment in inconclusive preoperative diagnosis.

## Highlights

• Renal leiomyoma is one of the least common benign renal tumors.
• Although most patients are asymptomatic, some may present with a palpable flank mass, abdominal or flank pain, and hematuria.
• Imaging may provide a clue with a general finding of a well-defined tumor margin and no local invasion.
• Preoperative imaging may be unreliable in some cases and histopathologic examination provides a definitive diagnosis.
• Radical nephrectomy remains as the mainstay of treatment in inconclusive preoperative diagnosis.

## 1 Introduction

Benign renal tumor incidence and detection rate is increasing in the last decade with the widespread use of abdominal ultrasonography and computed tomography (CT). Among all existing benign renal tumors, leiomyoma is one of the least common benign renal tumors. It is 0.3 % of all surgically treated renal tumors (both benign and malignant), and 1.5 % of all benign tumors [1]. Definite diagnosis is only through histopathologic examination. We report a case of a 43-year-old male presenting with hematuria and left renal mass treated with radical nephrectomy. The work has been reported in line with the SCARE criteria [2].

## 2 Case presentation

**Fig. 1:**
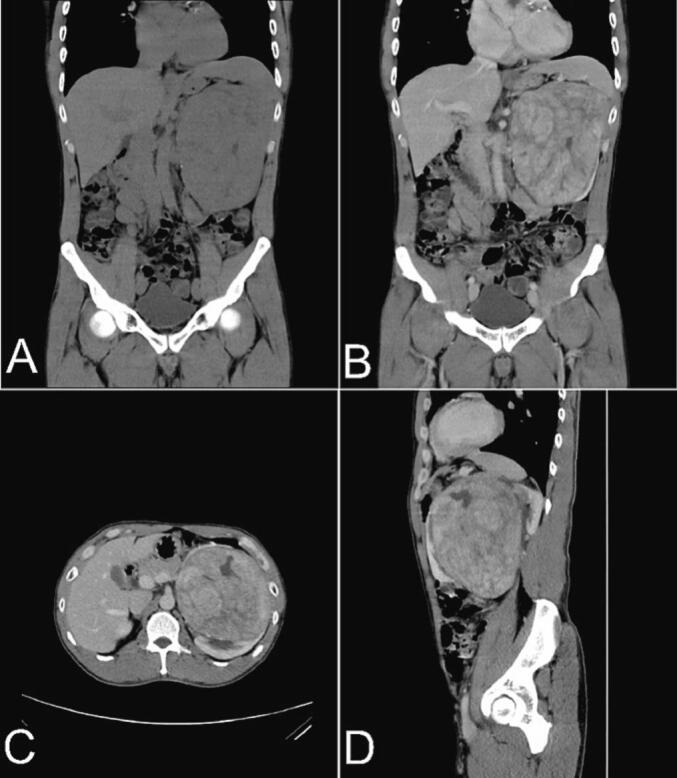
(A) Non-contrast Coronal view, (B) Coronal view with contrast, (C) Axial view with contrast, (D) Sagittal view with contrast.

A 43-year-old male was presented at the urology outpatient department with a chief complaint of palpable mass on the upper left abdomen. The mass had been progressively enlarged over the past 1 year. He felt mild abdominal discomfort and painless intermittent hematuria for the last 6 months. Contrast-enhanced CT scan revealed a solid heterogenous mass on the left kidney, predominantly on the anterolateral-caudal with the size of 11.3 cm × 11.8 cm × 15.1 cm which enhanced with contrast (Fig. 1). The mass was adherent to the left abdominal wall and pushed the spleen cranially. The patient's serum creatinine was 1.1 mg/dL.

**Fig. 2:**
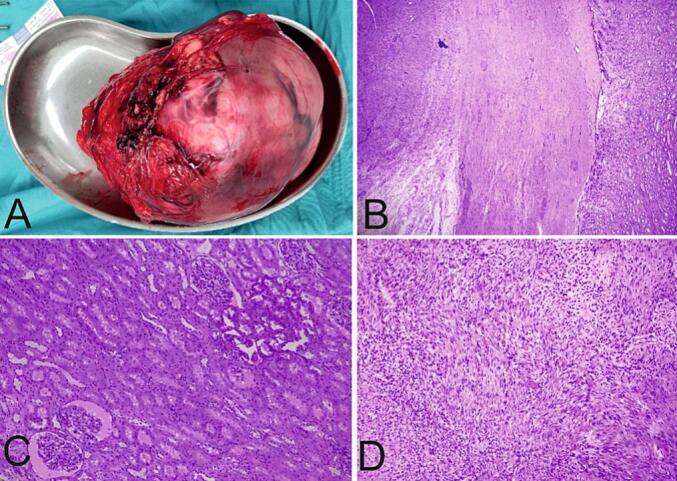
**(A)** Gross Section of tumor, **(B)** Hematoxylin-eosin (HE) staining 100× magnification, well demarcated tumor (black arrow), normal kidney parenchyma (white arrow), **(C)** Normal kidney, HE staining 200× magnification, **(D)** Tumor HE staining 200× magnification.

The patient underwent an open left radical nephrectomy and was discharged on postoperative day two. The excised tumor, measured about 15 cm × 11 cm (Fig. 2A). Histopathology results revealed leiomyoma (Fig. 2B-D). Two weeks after surgery, the patient was asymptomatic and his serum creatinine was 1.6 mg/dL.

## 3 Discussion

Leiomyoma is a rare benign renal tumor, found incidentally in 0.3 % of treated renal tumors [1,3]. Other studies stated that leiomyoma was found in 4–5.5 % autopsy [3]. Woman is the predominance of this case (2:1) and the average age of leiomyoma is 42-year-old [4]. Although most patients are asymptomatic, some may present with a palpable flank mass, abdominal or flank pain, and hematuria [3]. In our patients, all three symptoms were observed.

Macroscopically, the excised specimen was solid, well-circumscribed and non adherent to the surrounding organs. This was not consistent to pre operative imaging results where it was described that the mass was adherent to adjacent organs. Majority of renal leiomyoma were heterogenous due to its degenerative phenomena. [5,6] This may make leiomyoma difficult to differentiate with other benign lesions such as angiomyolipoma (AML) and oncocytoma and malignant tumors such as RCC and leiomyosarcoma. Imaging may provide a clue with a general finding of a well-defined tumor margin and no local invasion. In our case preoperative CT findings made the initial diagnosis inconclusive since it showed the renal mass was adherent to the abdominal wall. [6]

Definitive diagnosis was only possible through histopathologic examination. Histopathologic findings (Fig. 2A–D) showed proliferation of benign smooth muscle cells arranged within crossed fasciculi and there were parts which formed whorled-like appearance. There were no abnormal mitosis or coagulative necrosis found. These findings were consistent with another study by Brunocilla et al. [6]

Despite the preoperative radiological findings, the standard of care for a relatively large renal mass is a radical nephrectomy [6]. The prognosis of renal leiomyoma, after surgery, is excellent without recurrence [6,7]. In study by Gupta et al. [8], in 10 cases with clinical follow up, none had tumor recurrence. Although the neoplasm was benign, regular follow-up is essential to monitor symptoms and assess residual kidney function. This includes measuring kidney function through serum creatinine levels (as renography is unavailable at our hospital) and conducting abdominal imaging, such as ultrasound. In our patient, serum creatinine increased from 1.1 mg/dL to 1.6 mg/dL two weeks after surgery, despite a preoperative CT scan showing good contralateral kidney function. As noted in studies by Ellis et al. [9] and Qureshiet al [10], an increase in serum creatinine can be observed in male patients who underwent radical nephrectomy, potentially due to an increase in skeletal muscle mass. It is important to inform patients preoperatively about the risk of reduced kidney function (creatinine clearance) after nephrectomy, even if the contralateral kidney appears to function well.

## 4 Conclusion

Renal leiomyoma is a rare benign kidney tumor. Preoperative imaging may be unreliable in some cases and histopathologic examination provides a definitive diagnosis. Radical nephrectomy remains as the mainstay of treatment in inconclusive preoperative diagnosis.

## Consent

Written informed consent was obtained from the patient for publication of this case report and accompanying images. A copy of the written consent is available for review by the Editor-in-Chief of this journal on request.

## Ethical approval

Ethical approval was not required for this case reports in our institution, as case report are deemed not to be research.

## Funding

This research did not receive any specific grant from funding agencies in the public, commercial, or not-for-profit sectors.

## Author contribution

**Anak Agung Ngurah Krisnanta Adnyana:** writing–original draft, writing–review and editing. **Novitasari:** resources. **I Wayan Suarsana:** conceptualization, validation, writing-review and editing. **Ida Bagus Oka Widya Putra:** conceptualization, resources, validation, writing-review and editing, supervision. All authors approved the final version for publication.

## Guarantor

Ida Bagus Oka Widya Putra.

## Research registration number

Not applicable.

## Conflict of interest statement

None.
